# Evolution of Coronary Stents: From Birth to Future Trends

**DOI:** 10.3390/jcm15010047

**Published:** 2025-12-21

**Authors:** Zhuo Huang, Charles Skarbek, Yulin Li, Joseph Touma, Pascal Desgranges, Romain Gallet, Jean Sénémaud

**Affiliations:** 1 Laboratory for Vascular Translational Science, Inserm U1148, 75018 Paris, France; zhuo.huang@inserm.fr (Z.H.);; 2 Université Paris Cité, 75006 Paris, France; 3 AlchiMedics S.A.S., 75008 Paris, France; 4 Emergency Department, Pitié-Salpêtière Hospital, Assistance Publique—Hôpitaux de Paris, 75013 Paris, France; 5 Emergency Center, Zhongnan Hospital of Wuhan University, Wuhan 430071, China; 6 Department of Vascular Surgery, Henri Mondor University Hospital, Assistance Publique—Hopitaux de Paris, 1 Rue Gustave Eiffel, 94000 Créteil, France; 7 Faculté de Santé, Université Paris Est-Créteil, 94000 Créteil, France; 8 Department of Cardiology, Henri Mondor University Hospital, Assistance Publique—Hopitaux de Paris, 94000 Créteil, France

**Keywords:** coronary artery disease, percutaneous coronary intervention, coronary stents, stent coating, stent functionalization, personalized devices

## Abstract

Coronary artery disease (CAD) remains one of the leading causes of mortality worldwide, affecting more than 300 million people. Over the past two decades, percutaneous coronary intervention (PCI) has become the cornerstone of CAD treatment, involving the implantation of coronary stents. This review clarifies how coronary stents emerged, evolved, and ultimately reshaped modern interventional cardiology. Beginning with balloon angioplasty and progressing through bare-metal and drug-eluting stents, we show how each advancement solved key clinical shortcomings—dramatically reducing restenosis, thrombosis, and repeat revascularization. We further review the major technological advances driving modern stent development, such as biodegradable alloys and biomimetic coatings. We also highlight the remaining challenges, including long-term stability, manufacturing complexity, and limited translational readiness. Together, these elements support our central thesis: that the historical evolution of coronary stents is fundamental to understanding present PCI practice and to guiding the next phase of device innovation.

## 1. Introduction

Coronary artery disease (CAD) remains a leading cause of mortality worldwide, affecting 315 million people worldwide in 2022 [1]. The primary pathological mechanism of CAD is atherosclerosis, a progressive condition characterized by the accumulation of lipid-rich plaques and chronic inflammation within the vascular wall [2,3]. Over time, these plaques lead to stenosis, restricting blood flow and increasing the risk of major cardiovascular events such as myocardial infarction (MI) [4], stroke and lower limb loss, with significant rates of mortality and disabling complications in survivors. The landscape of atherosclerosis has changed, as it now involves younger patients and more women than before [5]. Over the past decade, the frequency of percutaneous coronary intervention (PCI) has risen by 62% in Korea and approximately 300% in China [6,7], with growth rates of 15.8% in the USA and 35.9% in Ireland [8,9]. In parallel, the global stent market is projected to grow at a compound annual growth rate of 3.7%, reaching $18.46 billion by 2030 [10], highlighting the need for sophisticated and durable arterial stent grafts.

Over the past few decades, coronary stents have undergone continuous innovation, with generations of refined structural designs, biocompatibility, drug-eluting properties, and overall clinical performance. This narrative review aims to explore the technical breakthroughs and their clinical impact on coronary stents throughout history, assessing how each technological advancement has shaped modern cardiovascular interventions. Furthermore, we discuss the current status and future directions in stent development, highlighting their potential to further enhance long-term outcomes and redefine the landscape of interventional cardiology in the setting of PCI expansion.

## 2. Methods

This review was conducted as a narrative literature review following the methodological recommendations of Green et al. and Sukhera J. [11,12]. The objective was to illustrate the historical development, technological evolution, and clinical implications of cardiovascular stents.

### 2.1. Literature Searching Strategy

A comprehensive search of the literature was performed between 1 January 1950 and 30 November 2025 using the following electronic databases: PubMed, Scopus, Web of Science and Google Scholar. Additional sources included major cardiology conference proceedings, FDA (U.S. food and drug administration) and EMA (European medicines agency) regulatory reports, industry white papers, and reference lists of key publications to identify relevant literature not captured by database searches.

Search terms were combined using Boolean operators and included variations of:“cardiovascular stent”, “coronary stent”, “PCI”, “PTCA”, “bare-metal stent”, “drug-eluting stent”, “DAPT”, “biodegradable scaffold”, “surface functionalization”, “stimuli”, “responsive”, “biomimetic”, “digital technology”, “coating”, “clinical outcomes”, “restenosis”, “thrombosis”, “long-term follow-up”, “cost-effectiveness”, and “regulatory approval”.

No language restrictions were applied.

### 2.2. Inclusion Criteria

Sources were included if they met the following criteria:

1. Addressed the design, function, evolution, clinical performance, or translational adoption of coronary or endovascular stents.
2. Reported original research, regulatory documentation, expert consensus, or real-world clinical data.
3. Contributed meaningfully to the historical, technological and clinical understanding of stents.

### 2.3. Exclusion Criteria

Studies were excluded if they were:

1. Focused exclusively on unrelated cardiovascular implants (e.g., valves, grafts).
2. Case reports with anecdotal value only.
3. Technical patents without supporting experimental or clinical evaluation.

### 2.4. Study Selection Process and Potential Selection Bias

Titles and abstracts were screened by the authors, followed by full-text evaluation when relevance was uncertain. Consistent with narrative review methodology, studies were selected on the basis of conceptual relevance rather than rigid hierarchical evidence scoring. To reduce selection bias, sources representing supportive, neutral, and contradictory evidence were all included when available.

## 3. Historical Development

### 3.1. Birth of PCI—A Glance into History (-1977)

Before Dr. Andreas Grüntzig performed the world’s first percutaneous transluminal coronary angioplasty (PTCA) in 1977, which laid the foundation for modern PCI [13], options were limited for patients presenting with CAD. The medical approach to managing coronary CAD involved lifestyle modification and drug treatment, aimed at alleviating symptoms and slowing disease progression [14,15]. These treatments were effective in relieving symptoms. However, they failed to address the underlying arterial blockage in severe CAD, particularly in patients with significant stenosis or acute coronary syndromes (ACS). Consequently, clinical outcomes were unfavorable. Coronary artery bypass grafting (CABG) was the only effective revascularization strategy, utilizing grafts from the saphenous vein, internal mammary artery, or radial artery to bypass occluded vessels [16]. Despite its efficacy in alleviating symptoms such as angina, CABG is a highly invasive procedure associated with prolonged recovery and considerable perioperative risks, including a 3–22% incidence of postoperative MI and a 5–22% mortality rate [17]. Consequently, there was a critical need for a less invasive yet equally effective alternative to surgical intervention.

#### Cardiac Intervention—A Long Journey

Although PCI has only a history of approximately 50 years, the idea of accessing central vessels using a dedicated catheter can be dated back to Werner Forssmann’s self-experiment in 1929 [18,19]. Driven by his bold idea of injecting medication into the heart, he inserted a urethral catheter through his left humoral vein and successfully captured images of it inside his own heart. This pioneering procedure was later advanced by André F. Cournand and Dickinson W. Richards, who used a catheter for intracardiac measurements. In 1956, these three scientists were jointly awarded the Nobel Prize in Physiology or Medicine [20]. Catheterization has become the standard method for measuring intracardiac pressure. However, at this time, the coronary arteries were still too intricate to tangle with.

A breakthrough happened in 1958 when F. Mason Sones accidentally injected contrast dye into a patient’s right coronary artery while inserting a diagnostic catheter into the ascending aorta. The patient experienced no discomfort, and a clear image of the coronary vasculature was obtained. This discovery led to the design of dedicated catheters with an open, tapered tip and sieve-like openings, which became prototypes for modern PCI devices [21].

After important refinement of the procedure by Melvin P. Judkins [22], coronary angiography soon became a safe and widely used method for diagnosing CAD. However, transluminal vascular procedures remained out of reach until Charles T. Dotter reported the first transluminal angioplasty procedure in 1964. Using a dilating catheter of his own design, he successfully recanalized a distal femoral artery occlusion through a femoral antegrade approach. The procedure was performed in a female patient with chronic limb-threatening ischemia and associated tissue loss. This achievement marked a breakthrough in interventional medicine [23]. This groundbreaking procedure was called “dottering”. This maiden intervention paved the way for balloon-assisted angioplasty, with the seminal work of Andreas Grüntzig, who developed a balloon on a catheter tip intended to be inflated. In 1974, he successfully performed his first balloon angioplasty on a 67-year-old man with iliac artery stenosis. Finally, in 1977, history was made—a 38-year-old man, refusing CABG, became the first to undergo coronary intervention with treatment of his left anterior descending artery using femoral access [24]. This procedure was named percutaneous transluminal coronary angioplasty, which marked the birth of PCI and the dawn of interventional cardiology.

### 3.2. PTCA to Stent—A New Era (1977–1986)

PTCA quickly gained global acceptance as a revascularization technique. However, with the steady increase in the number of cases performed, specific complications of this innovative technique, namely, restenosis and acute coronary thrombosis, were described [25,26]. After balloon expansion, the atherosclerotic plaque is compressed against the vessel wall, temporarily restoring the lumen diameter. However, this centrifugal compression does not prevent immediate renarrowing, owing to the vessel’s natural tendency to recoil. Moreover, excessive balloon inflation can cause varying degrees of endothelial injury, triggering thrombosis and inflammation, and in some cases, leading to vessel dissection and ultimately, acute thrombosis [27,28].

According to studies of PTCA patients around 1990, restenosis occurred in 20–50% of patients, with a 6-month reintervention rate as high as 20% [29], while acute thrombosis occurred in approximately 10% of patients [26]. These complications can result in recurrent angina, sudden cardiac death, and the need for repeated revascularization, significantly impairing the patients’ quality of life and long-term clinical outcomes.

#### Stent as Standard of Care

To address these complications, physicians have begun to conceptualize a permanent structural scaffold within the arterial lumen to overcome the limitations of PTCA. According to Dr. Ulrich Sigwart, he had this idea from the corrugated steel arches used to stabilize tunnel construction [30]. This effort began almost simultaneously in Europe and the USA, with Ulrich Sigwart and Jacques Puel developing self-expanding stents, while Julio Palmaz and Richard Schatz worked on balloon-expandable stents. In 1986, Ulrich Sigwart and Jacques Puel performed the first implantation of a self-expanding stent in the human coronary artery using the WALLSTENT^®^, a device constructed from stainless steel. The stent featured a braided mesh configuration composed of 16 wires, each with a diameter of 60 μm, and was delivered via a 3.0–3.3 mm catheter, constrained by a retractable plastic membrane. This landmark procedure was performed in Toulouse, France [31]. The following year, the first balloon-expandable stent, the Palmaz-Schatz^®^ stent, was introduced in the United States. Constructed from stainless steel, it featured a slotted-tube design with strut thickness ranging from 100 to 150 μm and was mounted on a 2.3–2.7 mm balloon catheter shaft [32]. Both effective as they proved to be, the implantation of self-expanding stents was more challenging due to the carrier system’s tendency to shift around the target site, making precise placement difficult [30]. This limitation is one of the reasons why balloon-expandable stents are preferred for most coronary interventions.

Although the invention of the bare-metal stent (BMS) was a milestone in interventional cardiology, they were not widely used until 7 years later. Early stents had thick struts and high metallic density, resulting in high thrombosis rates and deployment challenges [33]. Though reported rates of in-stent stenosis appeared lower than restenosis after PTCA, ranging from 17% to 41% [34,35,36,37,38], such restenosis remained a topic of debate among interventional cardiologists. The controversy continued until 1993, when the BENESTENT and STRESS trials provided the first high-quality evidence that BMS outperformed balloon angioplasty, establishing stenting as the standard of care [39,40]. The use of stents has grown exponentially since then. By 1999, at least 8 out of 10 PCI were performed with stenting [41].

### 3.3. Amelioration of Stenting—DAPT and Coatings (1986–2002)

As stenting has become more widely performed, specific complications of this procedure have begun to gain increasing notice. Cardiologists identified stent thrombosis (ST) and in-stent restenosis (ISR) as the primary post-procedure complications, along with rare issues such as bleeding, artery dissection, and stent infection (Table 1). ST is linked to a 50% incidence of acute myocardial infarction and has a 20% mortality rate [42], whereas ISR is the most common complication and often requires reintervention. Therefore, much effort has been made to address these issues, such as implementing dual antiplatelet therapy (DAPT) to lower thrombosis, coating anti-proliferative drugs on the stent to decrease hyperplasia within the lumen, and using new alloys with better mechanical and radiological performance. These advancements have significantly reduced the incidence of ST from rates as high as 20% to less than 1% [43], and ISR from up to 41% to approximately 2.5% with current-generation drug-eluting stents (DES) [38].

#### 3.3.1. Dual Antiplatelet Therapy

Following BMS implantation with single antiplatelet therapy during the 1980s to the mid-1990s, the rate of acute and subacute ST (within 24 h and 24 h to 30 days) was very high, ranging from 12% to 20% [42,44]. Therefore, aggressive treatment using different combinations of anticoagulant and antiplatelet drugs has been suggested. In the regimen of these treatments, the rate of ST was lowered significantly to 3–5%, but the risk of hemorrhage also leveled [39,40]. The first DAPT was introduced by Colombo et al. in 1993–1994 using ticlopidine for 1 month with short-term aspirin for 5 days after stent implantation [53]. They showed that with this DAPT method and adequate expansion of stents, a 1.6% incidence rate of ST could be achieved at 6-month follow-up without anticoagulation therapy. This therapy was explored further in subsequent years. Schomig et al. conducted a randomized comparison of antiplatelet and anticoagulation therapy after stenting in 1996. The results showed that DAPT (ticlopidine plus aspirin) lowered the risk of MI by nearly 80% and had a 7-times lower occlusion rate of the stented vessel compared to anticoagulant therapy (intravenous heparin, phenprocoumon, and aspirin) [54]. Additionally, no hemorrhagic complications were observed in the DAPT group within 30 days post-PCI. Moreover, advancements in pharmacology have led to the replacement of ticlopidine with clopidogrel, a P2Y_12_ receptor inhibitor that offers greater efficacy and improved tolerance. In 2001, the PCI-CURE substudy of the CURE trial demonstrated that DAPT significantly reduced cardiovascular death, MI, and urgent target-vessel revascularization in patients undergoing PCI [55]. In 2005, DAPT was officially recommended for patients undergoing PCI by the American College of Cardiology/American Heart Association (ACC/AHA).

Currently, DAPT remains the standard treatment for patients undergoing PCI. Especially, compared to clopidogrel, the utilization of the latest generation P2Y_12_ inhibitors (ticagrelor, prasugrel) has reduced even more the rate of ST by about 30% [56]. However, with the evolution of cardiovascular stents, their duration has become increasingly personalized. Whereas a fixed duration of more than 12 months was recommended in 2005, recent guidelines, particularly since 2020, support strategies as short as 1 month of DAPT followed by aspirin monotherapy or a P2Y_12_ inhibitor alone in selected patients at high risk of bleeding [57,58,59]. However, these recommendations are largely derived from trials in carefully selected high-bleeding-risk populations treated with contemporary DES, and long-term data are still limited. Therefore, 12-month DAPT remains the default for many patients and the real clinical benefit of ultra-short regimens in broader real-world practice remains uncertain.

#### 3.3.2. Stent Coating—A Revolutionary Concept

Coating represents a major theoretical breakthrough in stent development. As clinicians increasingly recognized that stents act as foreign bodies within the arterial wall and bloodstream, attention turned to their role in triggering adverse biological responses. Thrombosis occurs due to platelet adhesion and leukocyte activation, and neointimal hyperplasia is driven by excessive smooth muscle cell (SMC) proliferation [28,60]. To mitigate these complications, it became evident that a protective “shield” layer is needed to insulate tissues and cells from direct contact with the stent surface.

Stent coating refers to a thin layer of material applied to the surface of a stent to enhance its therapeutic effect, including better biocompatibility, reduced neointimal hyperplasia, and less thrombogenicity. Today, DES has a class 1 recommendation over BMS for all patients and is specified as standard during PCI for acute coronary syndromes [61,62]. Before their use in cardiovascular stents, coatings were initially used in stents designed for other medical conditions, such as ligament repair, tracheal obstruction, and biliary disease [63,64,65]. However, unlike vascular stents, these implants are not exposed to circulating blood, making their biological environment less complex than that of coronary stents.

The material and function of the coating vary according to the intended purpose, allowing for high creativity and innovation in design. In this article, coatings are divided into three categories according to their historical development: biocompatible coatings, drug-delivery coatings, and polymer-free coatings/surfaces [66]. Nevertheless, these three stages are not strictly distinct but overlap to some extent, reflecting the continuous evolution and refinement of stent coatings.

#### 3.3.3. Biocompatible Coatings—Early Attempt

The idea of a biocompatible coating originated from the thrombus formation and corrosion of stainless steel used to construct the BMS. Upon implantation, ions released from the stent trigger inflammation immediately, causing platelet adhesion and aggregation, eventually leading to ST [67]. Such detrimental interactions between the metallic stent structure and blood components support the concept of stent modification. However, owing to technical limitations in the early 1990s, achieving a balance between mechanical behavior and biocompatibility was difficult.

There are mainly two ways to fabricate coatings: the passive method, by polishing the surface with inert material like silicon carbide [68], gold [69], or polyurethanes [70]; or an active method, by using active compounds to reduce thrombosis, including prostacyclin [71] and heparin [72]. There are also more advanced bioactive coatings, such as endothelial cell-seeded stents or extracellular matrix-coated stents [73]. They are designed to enhance endothelialization, or fibrin-coated stents [74] aimed at bypassing the thrombosis process since polymerized fibrin is not thrombogenic. In animal experiments, many of these coatings have been reported to reduce ST compared to BMS. However, failing to efficiently address the intricate underlying pathological mechanisms of ISR [60], none of these coatings significantly reduced ISR or improved clinical outcomes [75,76,77]. Additionally, with the widespread adoption of DAPT for successful ST control, most of these approaches have been abandoned. Nevertheless, these attempts laid an important foundation for modern and future stent design, such as the use of polymers and bioactive components, which provided experience for further breakthroughs.

#### 3.3.4. DES—A Revolutionary Drug-Delivery Coating

The previously described coating strategies failed to improve clinical outcomes. They were associated with high ISR rates and smaller minimal luminal diameters. Moreover, they failed to reduce adverse coronary events compared with BMS [76,77]. At the same time, a revolutionary coated stent—drug-eluting stent, was on the horizon. The major mechanism of ISR is hyperplasia of SMC. Therefore, many antiproliferative and cytotoxic drugs have been incorporated into polymer carrier systems, which were then coated onto the stent for localized drug delivery. Drugs such as actinomycin-D (an antitumor antibiotic), colchicine (a microtubule inhibitor), methotrexate (an anti-metabolic agent), and the cortical hormone dexamethasone have been tested [78,79]. However, despite promising results in vitro and in vivo, none of these drugs has shown good efficacy in reducing ISR in clinical trials [80,81].

The first effective drug to reduce ISR in humans was sirolimus, an immunosuppressive compound that inhibits the mTOR pathway, blocking cell cycle progression from the late G1 to S phase, thereby suppressing SMC proliferation [82]. Another promising drug was paclitaxel, a well-known anticancer drug that inhibits cell division and proliferation [83]. These drugs were loaded into a polymer and coated on the surface of BMS for continuous release for a few weeks after implantation. The sirolimus-eluting stent (Cypher^®^, Cordis, Hialeah, FL, USA) received CE Mark approval in 2002 and FDA approval in 2003, followed by the paclitaxel-eluting stent (Taxus^®^, Boston Scientific, Marlborough, MA, USA) the next year. Clinical trials confirmed the efficacy of these two DES, demonstrating that Cypher^®^ stents reduced ISR from 36.3% with BMS to 8.9% [84], while Taxus^®^ stents lowered ISR from 26.6% to 7.9% [85]. These revolutionary stents soon became widely used because of their superiority in reducing ISR and improving patient outcomes. By 2005, more than 90% of stents implanted in the USA and Europe were DES [86].

## 4. Current Stent Technologies

### 4.1. A Rapid Evolution (2002–Now)

**Figure 1:**
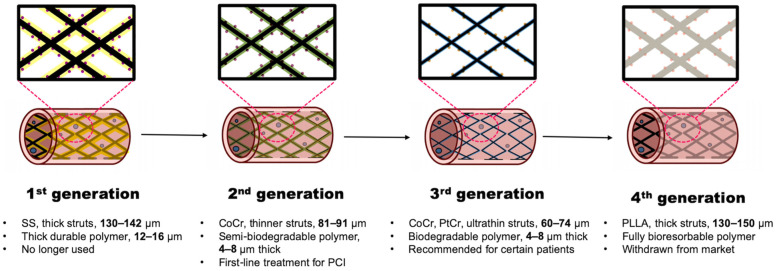
Key features of 1st–4th generation DES. Black and gray bars represent metal and polymer scaffolds, respectively. The colored bars represent the coatings. The small dots represent drugs carried by the polymer. Clinical recommendations were obtained from the 2023 ESC guidelines.

Considering the clinical efficacy of DES, one might assume that most challenges associated with stent implantation have been resolved, leaving little room for further improvements. However, this was not entirely accurate. With the widespread adoption of DES, a new complication, late stent thrombosis (LST), has emerged. Previously, ST was considered an early stage issue, mostly occurring within 30 days of implantation. However, LST can develop months or even years later, posing significant clinical risks. In 2004, McFadden et al. [87] reported four cases of LST in patients implanted with paclitaxel-eluting (343 and 442 days) or sirolimus-eluting (335 and 375 days) stents soon after the interruption of antiplatelet therapy, which eventually caused MI. The relationship between DES and LST was further confirmed in the following studies [88,89]. Such LSTs were explained by delayed endothelialization by anti-proliferative drugs and inflammatory reactions to polymers, leading to platelet aggregation and ST. Consequently, DES use declined between 2006 and 2008, following the European Society of Cardiology (ESC) congress 2006, where Camenzind et al. highlighted the increased risk of late thrombosis [90]. However, this also prompted technological advancements in stent design and stimulated the development of novel antiplatelet drugs. Over the past two decades, DESs have undergone four generations of continuous refinement, representing modern stent advancements [91]. Following the evolution from first- to fourth-generation DES, this chapter provides an overview of the current stent technologies (Figure 1).

#### 4.1.1. First-Generation DES

As described above, first-generation DESs mainly feature the incorporation of effective anti-proliferative agents (sirolimus and paclitaxel) and the use of durable polymers for sustained drug release. They achieved great efficacy in reducing ISR and replaced BMS in most implantations. However, this generation of DES had thick struts and permanent drug-delivery polymers (Table 2), which were incriminated in the cases of LST. In 2006, the BASKET-LATE trial [92] revealed that stopping DAPT before 12 months in DES patients led to higher rates of LST than BMS. Therefore, in the same year, the ESC and AHA began recommending at least 12 months of DAPT for first-generation DES to reduce the risk of LST, although this extended therapy also increased the risk of bleeding [93]. These defects led to further refinements in the next-generation DES.

#### 4.1.2. Second-Generation DES

The first second-generation DES was Endeavor^®^ (Medtronic), which gained CE Mark approval in 2005, followed by Xience V^®^ (Abbott) in 2006 and Resolute^®^ (Medtronic) in 2008. There were three major improvements compared with first-generation DES.

First, structural refinement was performed using alloys such as cobalt-chromium (CoCr) to replace 316 L stainless steel (SS). CoCr has a stronger mechanical strength than SS, allowing for thinner struts with a lower risk of ISR [99] and enhanced flexibility. It also offers greater biocompatibility and enhanced radiopacity, making it more durable and allowing better visualization during implantation [100]. Currently, CoCr or platinum-chromium (PtCr) has replaced SS in most second- and third-generation DES.

The second refinement involves the incorporation of an enhanced and thinner polymer. Polymers used in first-generation DES, such as poly(ethylene-co-vinyl acetate)/poly(n-butyl methacrylate) (PEVA/PBMA) and styrene-isobutylene-styrene (SIBS), were thick, durable, and thrombogenic, causing delayed endothelialization and continued inflammatory response. The drug release pattern of these polymers was also unstable, with Cypher^®^ releasing 80% sirolimus in 30 days, while Taxus^®^ featured a rather slow release mode. Therefore, in second-generation DES, a highly biocompatible fluoropolymer was used in Xience V^®^, which was significantly thinner and featured a hydrophobic surface. As a result, the risk of LST in second-generation DES was approximately 80% lower than that in first-generation DES [101]. Another hybrid polymer system, BioLinx^®^ (Medtronic), which combined biodegradable and durable polymers, demonstrated safety and long-term clinical outcomes comparable to other second-generation DES. It also showed non-inferior efficacy compared with third-generation DES [102,103]. Importantly, these polymers offered a smoother and more sustained drug release profile, enhancing the controlled and prolonged anti-restenotic effect.

The last vital improvement was the use of novel anti-proliferative agents. With the development of pharmaceutical technology, paclitaxel was gradually abandoned because of its unstable release, lower efficiency, and inferior safety compared to the ‘limus’ family of drugs [95]. In clinical practice, the ‘limus’ family remains the sole class of coated drugs at the coronary level, owing to their optimal balance of efficacy, safety, endothelial compatibility, and controlled-release kinetics. In this generation of DES, new sirolimus derivatives, such as zotarolimus and everolimus, have been widely applied. Zotarolimus is a semisynthetic derivative of sirolimus, with a tetrazole ring substitution at position 42, replacing the hydrophilic hydroxyl group. This modification increases lipophilicity, making it the most water-repellent sirolimus analog. Consequently, zotarolimus exhibits sustained, slow-release kinetics rather than burst release [104]. Everolimus, another sirolimus derivative, is widely regarded as the best-performing drug among second-generation DES. It features a 2-hydroxyethyl substitution at position 40, which enhances polarity and lipophilicity. Compared with sirolimus, everolimus offers better bioavailability, longer cellular retention, reduced clearance, and improved absorption [105].

Currently, second-generation DES have largely replaced first-generation DES. Many trials have proven their superiority in reducing LST and promoting long-term outcomes [95,106,107]. Despite the rapid development of new technologies, they remain the standard of care for PCI patients in guidelines worldwide.

#### 4.1.3. Third-Generation DES

Third-generation DESs were introduced between 2012 and 2014, with the aim of further improving clinical outcomes in patients undergoing PCI. This generation of DES features ultrathin struts and, most importantly, biodegradable polymers, which break down gradually into compounds that can be metabolized naturally. The use of these polymers has brought another revolution to DES, reducing the risk of polymer-induced inflammation and LST, thus shortening the duration of DAPT and ameliorating patient outcomes. This advancement has expanded their suitability for patients with complex clinical conditions, such as high bleeding risk and diabetes [108]. The major third-generation DES currently used in clinical treatment include the Synergy^®^ (Boston Scientific), Orsiro^®^ (Biotronik), and YukonPC^®^ (Translumina, Hechingen, Germany) stents.

The Synergy^®^ stent is built on an ultrathin PtCr (74 µm) platform and biodegradable poly (lactic-co-glycolic acid) (PLGA) polymer as a drug carrier (Table 2). PLGA is a copolymer of lactic and glycolic acids. Upon exposure to water, PLGA gradually degrades into lactic acid and glycolic acid monomers, which are subsequently metabolized into carbon dioxide and water or converted to glyoxylate and excreted renally. The PLGA coating was also ultrathin, with a thickness of only 4 µm. Full drug release is achieved in approximately 90 days, with complete PLGA degradation shortly thereafter [109]. The efficacy of the Synergy^®^ stent has been proven in some clinical trials, such as the EVOLVE and EVOLVE II trials, which showed non-inferior results for Synergy^®^ compared to second-generation DES [110].

The Orsiro^®^ stent features a hybrid dual-coating design, with a CoCr platform and ultrathin 60 µm struts. It is first coated with a passive silicon carbide layer (PROBIO^®^) to enhance biocompatibility, followed by an active coating of poly l-lactic acid (PLLA) polymer and sirolimus (BIOlute^®^). The coating thickness is approximately 7.5 µm, with 50% of the drug released within the first 30 days, and the BIOlute^®^ polymer is fully degraded over 12–14 months. Orsiro^®^ showed non-inferior outcomes compared to Synergy^®^ and Resolute^®^ in the BIO-RESORT trial [111] and demonstrated superior efficacy compared to Xience Prime^®^ (Abbott) in the BIOSCIENCE trial [112].

Another category of third-generation DES is polymer-free DES, which uses the metallic stent surface itself as a drug reservoir, eliminating the need for polymer coatings. This concept is appealing because it avoids complications such as chronic inflammation and LST, which are associated with polymers. However, this kind of DES faces critical challenges in terms of drug adhesion and controlled release. To overcome these issues, efforts have focused on surface modification techniques that can transform BMS into drug reservoirs [113]. This is typically achieved by engineering grooves, nanopores, or macropores into the stent struts using high-precision manufacturing or by spraying crystallized drugs directly onto the surface. Such devices include the NEVO^®^ (Cordis), Janus^®^ (Sorin Biomedica), and Yukon^®^ (Translumina) stents. Despite their promise, the lack of robust clinical evidence has limited their widespread adoption. Their efficacy and long-term outcomes remain to be studied.

#### 4.1.4. Fourth-Generation DES

The concept of fourth-generation DES centers on the bioresorbable vascular scaffold (BVS), which is designed to gradually degrade and fully disappear from the vessel, leaving no permanent implant at the target lesion site. The first and most well-known BVS was the Absorb^®^ (Abbott) stent, which entered the market in 2011. Constructed from PLLA with a 150 µm thick strut, combining a poly(d,l-lactic acid) (PDLLA) based drug carrier coating, the scaffold is fully resorbed over approximately 3 years. Although the concept held great promise, the results of the ABSORB clinical trials were disappointing [98]. Compared to second-generation DES, Absorb^®^ showed higher Late lumen loss (LLL) and an increased incidence of ST. This was probably due to its thick structures and insufficient strength. The strut thickness of PLLA scaffold was 150 µm, about 2 times of most second and third-generation DESs. Thick struts can cause disturbed laminar flow, delayed endothelialization and increased platelet activation, leading to higher device thrombosis and late lumen loss. According to a randomized study, compared to thick struts (140 µm), stents with thin struts (50 µm) reduced the risk of restenosis by 42% in 1 year with significantly lower LLL [114]. Also, as a polymer, PLLA has a tensile modulus that is 100-fold lower than CoCr or SS, which led to its inferior radial expansion under the same load [115].

These disadvantages ultimately led to its withdrawal from the market in 2017. Nevertheless, this technology is not yet obsolete. In fact, new-generation BVS is now increasingly used in peripheral artery disease below the knee and has shown promising outcomes [116]. With the development of more advanced scaffolds, we have a good reason to expect the return of BVS for the treatment of CAD in the future (Figure 1).

### 4.2. Personalized DAPT

The rapid development of newer generations of DES has also changed DAPT for patients undergoing PCI. In the case of first-generation DES, patients were normally suggested for prolonged DAPT (>12 months) to avoid LST. This was effective but dangerous for those with high bleeding risk, which accounts for approximately 10% to 40% of PCI patients [117,118]. Following the adoption of second- and third-generation DES, the typical duration of DAPT has been reduced to 6–12 months and 3–6 months for stable CAD, respectively, owing to their improved safety profiles. In selected patients, particularly those with a high bleeding risk, DAPT may even be safely shortened to less than 3 months [119]. However, the optimal DAPT strategy should be individualized, considering the patient’s overall health, clinical indications, and specific stent type implanted.

## 5. Future Directions

**Figure 2:**
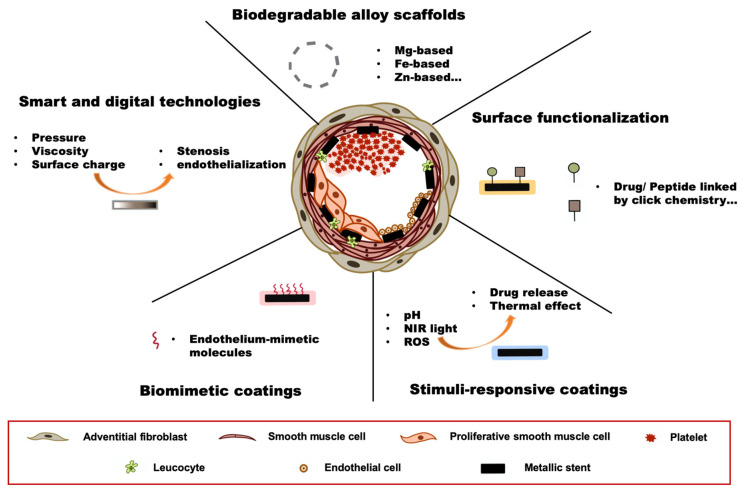
Outcomes of cardiovascular stents (**center**) and future directions of stent technology (surrounding area). Following ideal stent implantation, the regenerated endothelial cells provide complete neointimal coverage (**center**, **lower right**). However, when stents are coated with antiproliferative drugs or durable polymers that induce chronic inflammation, endothelialization is delayed (**center** and **upper right**). The two major complications of stent implantation are stent thrombosis, characterized by platelet aggregation and fibrin deposition (**center**, **upper left**), and in-stent restenosis, caused by excessive proliferation of smooth muscle cells (**center**, **lower left**). Future stent technologies and their functionalities are listed below.

With advancements in stent technology and adjunctive therapies over the past two decades, PCI efficacy and safety have improved remarkably. However, this progress has not slowed down innovation. In contrast, stent development remains a highly interdisciplinary field driven by breakthroughs in materials science, pharmacology, bioengineering, and biotechnology. These stents target not only the reduction in classic issues such as ST and ISR, but also the minimization of foreign body reaction, promotion of endothelialization, and achievement of individualized treatment. In this section, we highlight promising directions that are shaping the next generation of coronary stents (Figure 2).

### 5.1. Biodegradable Alloy Scaffolds

The primary limitation of early bioresorbable scaffolds is their poor mechanical strength, largely due to their polymeric composition, such as PLLA. To address this issue, researchers have actively explored alternatives that offer both structural integrity and controlled biodegradability. One promising direction is the development of biodegradable alloys. Composed of physiologically essential elements such as magnesium (Mg), iron (Fe), and zinc (Zn), these alloys gradually degrade in the body without causing adverse effects [120].

The Magmaris^®^ (Biotronik) stent, the first magnesium-based bioresorbable drug-eluting stent, is composed of WE43 magnesium alloy (Mg-Y-RE-Zr) and features a PLLA coating as a sirolimus carrier. Compared with polymer-based scaffolds, it offers improved biocompatibility and superior mechanical strength [121]. The scaffold and its coating degrade gradually over 12 months, ultimately restoring the natural structure of the vessel. In the BIOSOLVE-II trial, Magmaris^®^ demonstrated favorable long-term safety and clinical performance, with consistently low target lesion failure rates and no reported cases of definite or probable scaffold thrombosis over a 5-year follow-up [122]. However, some studies of first-generation magnesium scaffolds demonstrated rapid loss of radial strength and marked late lumen loss, which led to very high reintervention rates, with target-lesion revascularization approaching 24% at 4 months and 45% at 1 year [123,124]. These findings highlighted the need for a thorough evaluation of the safety and long-term mechanical stability of this new class of scaffolds.

Fe-based alloys offer excellent mechanical strength and radial forces. However, their slow degradation and accumulation of iron oxides in vascular tissues may hinder healing and impair regeneration [125].

Zn-based alloys have emerged as promising candidates for biodegradable scaffolds. It is the second most abundant element in the human body and plays a vital role in many biological processes. Zn-based alloys offer a moderate degradation rate (1–2 years) and possess greater mechanical strength than Mg [126]. Studies have demonstrated their good hemocompatibility and antiatherogenic properties [127,128]. However, their long-term vascular safety remains unclear. Other metals, such as calcium (Ca), lithium (Li), and molybdenum (Mo), are also being explored for future biodegradable scaffolds because of their distinctive physicochemical and biological properties.

### 5.2. Surface Functionalization

Surface functionalization refers to the process of modifying a material’s surface to introduce specific chemical and physical properties. In stent technology, this is typically achieved through plasma treatment, chemical etching, or surface structuring, which introduces reactive groups or alters surface properties. These modifications enable subsequent polymer grafting or the attachment of bioactive molecules, thereby enhancing stent performance.

Recently, with the development of material science and chemistry, more advanced surface functionalization methods have emerged, notably the use of polydopamine (PDA), click chemistry, and bio-orthogonal chemistry [129]. In 2007, inspired by mussel adhesion, Lee et al. found a method using dopamine self-polymerized to form an ultrathin (~45 nm), adhesive and bioactive PDA layer to almost any material surface [130]. PDA coating has excellent biocompatibility [131], showing no damage to multiple organs and cells in vitro [132]. Additionally, the PDA layer is rich in -NH_2_ and -OH groups that can conjugate with various peptides and drugs, enabling great potential for coating design. Cheng et al. coated nanoparticles with a PDA film and conjugated them with folic acid to treat cancer cells, and in vitro experiments showed an enhanced tumor-targeting effect [133]. Tiwari et al. reported that a PDA-coated nanofiber could enhance targeted photothermal therapy [134]. Although PDA coatings offer versatility, limitations such as uncertain hemocompatibility, lack of standardization, and insufficient long-term safety data have hindered their clinical translation [132,135].

Click chemistry is a rapid and selective chemical strategy that enables efficient molecular assembly under mild conditions, as exemplified by copper-catalyzed azide-alkyne cycloaddition. Biorthogonal chemistry extends this concept to living systems, allowing reactions to occur without disrupting native biology [136]. These methods enable fast, selective, and biocompatible surface modifications, facilitating the conjugation of biomolecules without interfering with native biological processes. In the past two decades, these technologies have been widely used for surface functionalization in living organisms. Zhang et al. developed a two-step strategy combining catechol functionalization with thiol-ene click chemistry for robust and versatile modification of metal surfaces [137]. Yang et al. introduced a strategy to functionalize metal stents by first coating them with an azide-modified mussel-inspired peptide, followed by grafting dibenzocyclooctyne (DBCO)-modified bioactive molecules. This dual-functional coating enabled the release of nitric oxide (NO) and the capture of endothelial progenitor cells. It showed strong antithrombotic effects, inhibited smooth muscle proliferation, and enhanced endothelialization, effectively preventing ISR [138]. However, when applied on a solid surface, the diffusion and accessibility of click chemistry regents should be further validated, as well as the long-term stability of the linker [139].

### 5.3. Stimuli-Responsive Coatings

Stimuli-responsive coatings are designed to alter their physical, chemical, or biological properties in response to specific external stimuli, such as pH, temperature, light, enzymes, and redox conditions. For coronary stents, such dynamic coatings can enable on-demand drug release, anti-thrombogenic switching, and environment-sensitive degradation, offering control over healing processes and reducing complications such as ST and ISR.

During stent placement, mechanical damage to the arterial walls induces inflammation, leading to an acidic microenvironment (pH 6.0–7.0) and accelerated atherosclerosis [140]. Therefore, several pH-responsive drug delivery systems have been developed to counter its progress. Xu et al. reported a drug delivery system based on electrostatic interactions, which released 90% of the drug at pH 6.0 within 48 h, but released less than 50% at pH 7.4 [141]. Ma et al. introduced another system based on pH-sensitive benzoyl imine linkages, which could release 90% of paclitaxel within 48 h at pH 5.5, compared to only 50% at pH 7.4 [142]. These technologies offer a controlled release of therapeutics to treat stent complications more effectively.

Light-responsive coatings, particularly those activated by near-infrared (NIR) light, have been explored for controlled drug delivery and activation in biomedical applications. NIR light, typically in the wavelength range of 700–1000 nm, offers excellent tissue penetration, minimal photodamage, and spatial-temporal controllability. Incorporating photothermal or photosensitive agents into stent coatings enables NIR-triggered local heating or chemical activation for controlled drug release, antibacterial effects, and surface property modulation.

Singh et al. coated gold nanorods on the surface of a BMS to generate heat upon NIR laser exposure. After implantation in the rat carotid artery, this technology proved to be effective in restoring blood flow within the occluded vessel by ablating the thrombus within the lumen [143]. Wang et al. reported an NIR-activated PLGA microsphere that achieved the on-demand release of drugs [144]. Other applications, such as shape memory polymers, have also been explored using NIR activation [145].

Another target is reactive oxygen species (ROS), which are overproduced after stent implantation due to local vascular injury, and contribute to inflammation, endothelial dysfunction, and ISR [146]. To address these challenges, ROS-responsive coatings have been developed to provide targeted therapeutic responses to oxidative stress. For instance, Wang et al. engineered a coating composed of epigallocatechin gallate and cysteine hydrochloride on 316 L SS stents. This coating, cross-linked with disulfide bonds, responded to elevated ROS levels by accelerating the release of pitavastatin calcium, effectively inhibiting inflammation and reducing the risk of restenosis in vivo [147]. Similarly, Chen et al. developed an ebselen-based ROS-responsive coating that exhibited antioxidative properties, promoted endothelialization, and prevented hyperplasia, thus enhancing the biocompatibility of cardiovascular stents [148]. These strategies enable site-specific drug release, improving the efficacy of stents while minimizing systemic side effects of the drugs.

Although stimuli-responsive coatings offer the potential for on-demand drug release and adaptive behavior that could enhance vascular healing, their translation to coronary stents remains challenging. The complex, high-shear vascular environment may impair reproducible triggering. The increased material complexity also introduces risks such as uneven activation, coating delamination, or outright failure. In addition, long-term data on durability, hemocompatibility, and degradation under physiological conditions remain limited [149].

### 5.4. Biomimetic Coatings

Biomimetic coatings refer to surface modifications that replicate or simulate the structure, function, or biological activity of natural tissues, particularly the vascular endothelium, to avoid immune detection and inflammation. Healthy vascular walls perform physiological functions, such as antithrombogenicity, anti-inflammation, and regulation of vascular tone (such as NO release). By incorporating proteins, peptides, polysaccharides, or enzymatic systems, biomimetic coatings are expected to simulate a bioactive surface between the stent and surrounding tissues.

Several biomimetic strategies have been developed to endow stent surfaces with bioactive functions. Research team of Caligiuri, G developed an endothelial-mimetic coating by grafting a CD31 agonist peptide on metal stents, a key mediator of endothelial and leukocyte homeostasis and arterial healing. In a porcine model, this coating promoted rapid endothelialization within 7 days of implantation without triggering platelet or leukocyte activation. Compared with bare-metal and drug-eluting stents, CD31 agonist coating resulted in reduced thrombosis and more favorable neointimal remodeling [150]. This approach stimulated further investigations into CD31-mimetic peptides and coatings, opening new avenues for improving the performance of cardiovascular implants [151]. In another study, Zhao et al. constructed an NO-releasing coating by immobilizing a copper-based catalytic complex that mimicked endogenous NO production. This system provides sustained NO release, preventing thrombosis and promoting vascular healing [152]. Qiu et al. introduced a coating that combined heparin and NO-generating compounds. This coating inhibited platelet adhesion and smooth muscle proliferation while promoting endothelial cell growth, enhancing stent endothelialization, and lowering the risk of ISR [153]. Despite their promise, these strategies are still largely at the proof-of-concept stage, and rigorous evaluation of their long-term stability and in vivo safety is still needed.

### 5.5. Integration of Smart and Digital Technologies

With advances in microelectronics and biosensing technologies, increasing efforts have been made to integrate digital devices into the stents. These “smart stents” enable real-time monitoring of the local vascular environment and provide feedback to patients and clinicians alike. Typically, the monitored information includes blood pressure, pulse, and blood flow. Some sensors can detect endothelialization and restenosis [154,155].

Pressure changes within the vessel reflect obstruction or stenosis, making pressure sensors a common tool for monitoring stent patency. Chow et al. developed a stent capable of analyzing pulmonary arterial pressure by calculating tissue-induced power loss. However, the antenna and conductive rings on its surface may cause complications during PCI [155]. Researchers from team Takahata, K proposed the concept of using the stent platform itself as a wireless transmission component. In this design, a capacitive pressure sensor responds to local hemodynamic pressure changes as a variable capacitor, whereas the SS stent structure functions as an electrical inductor or antenna for wireless signal transmission [156]. Recently, ultra-thin and flexible pressure sensors based on polyimide-carbon nanotube composites have been applied to stents, offering a wide sensing range (10–500 kPa) and enhanced long-term stability [157].

Wireless passive magnetoelastic sensors fabricated from iron-based metallic glass ribbons. These devices detect mass loading or viscosity changes by monitoring shifts in the resonant frequency when exposed to an alternating magnetic field [158]. These features can be used to detect neointimal proliferation when sensors are applied to the interior walls of stents [159]. Self-sensing piezoelectric sensors based on microcantilevers have also been employed to monitor stent endothelialization. Upon contact with endothelial cells, changes in the effective mass and surface charge alter the resonance frequency of the cantilever, providing real-time information on endothelial coverage on the stent surface [154]. Recently, nanosensors and NO detectors have emerged as promising approaches for the precise monitoring of stent endothelialization [160].

Despite their theoretical advantages, there have been reports of the instability of long-term in vivo monitoring [161]. Also, their complex design poses challenges for the manufacturing process.

## 6. Discussion

The evolution of coronary stents is one of the greatest achievements in cardiovascular medicine, demonstrating the transformative power of multidisciplinary innovation. However, it’s important to look into not only the advantages of these technologies but also their limitations, as they are essential for real-world practice.

Introduced in 1977, PTCA offered a less invasive alternative to CABG with high acute procedural success and rapid symptom relief. However, its long-term clinical impact was limited by high restenosis and repeat revascularization rates. Randomized comparisons with CABG in multivessel disease showed broadly similar 10-year survival between PTCA and CABG overall (71% vs. 73.5%), but consistently much higher rates of repeat revascularization after PTCA (76.8% vs. 20.3% with CABG) and more recurrent angina, especially in diabetics and complex disease [162]. Economic analyses found that the initial hospital cost of PTCA was roughly half that of CABG, but by 2–3 years, cumulative costs converged because of frequent repeat procedures [163]. Despite these limitations, the availability and efficiency of PTCA in ACS still led to its fast expansion in clinical practice. According to a survey in Finland, in 1994–2013, 85,482 PCIs and 74,338 CABGs were performed. During this period, PCI rates quadrupled while CABG rates declined by two-thirds [164]; this trend set the stage for the later stent era.

The invention of stents addressed the main limitations of PTCA, namely acute vessel recoil, dissection, and abrupt closure, by providing a rigid scaffold to keep the artery open. Nevertheless, long-term data confirm that BMS use is associated with substantial late events. A 10-year follow-up cohort in Japan showed that the survival of BMS-implanted patients was 73.6% but event-free survival dropped to 39.2%, indicating the high rate of revascularization [165]. In another 3-year follow-up study from Sweden, BMS group showed restenosis rates of 15–20% and re-intervention rates of 5–10% [166].

The emergence of first-generation DES offered promising solution for these complications, taking over the share of BMS rapidly in few years. However, in mid to long-term follow-up studies, the safety and efficacy of these DESs was questioned. Although showed similar mortality in 3 years, patients with DESs had a significantly higher event rate, with 12.7 more events per 1000 patients per year after 6-month implantation, as well as more frequent very late stent thrombosis (adjusted risk ratio (RR): 2.89, 95% confidence interval (CI): 1.48–5.65) [166,167]. These defects drove the improvement of DES into second-generation, which showed better long-term safety and effectiveness. At five-year follow-up in the ENDEAVOR II trial, several clinical endpoints favored the Endeavor^®^ zotarolimus-eluting stent. Rates of target vessel failure were lower (15.4% vs. 24.4%), as were rates of target vessel revascularization (10.7% vs. 20.1%). Major adverse cardiac events were also reduced (15.4% vs. 24.6%). In addition, target lesion revascularization occurred less frequently (7.5% vs. 16.3%). Overall, the Endeavor^®^ stent demonstrated significantly better outcomes than BMS [168]. However, according to a study in Australia, the average cost of DES is about $800 each, while DES about $3300 each [169]. A 5-year cost-effectiveness model from Europe showed that modern DES could reduce overall cost by €184 compared with BMS over 5 years, while improving cardiac event-free survival by 0.54 years [170]. Such huge gap of price and limited health benefit poses difficulties in clinical practice, especially in developing countries. As for third-generations of DESs, although there have been several studies concluding their efficacy, there is limited data to validate their overall superiority against second-generation DES, nor their share of market.

Despite major advances in stent design, several clinical scenarios continue to challenge contemporary PCI. Patients with diabetes mellitus exhibit more diffuse atherosclerosis, impaired endothelial healing, and excessive neointimal proliferation [171]. Even with modern DES, they have higher rates of ST-elevation MI, non-ST-elevation ACS and stable CAD (23.2%, 17.8% and 12.7% for diabetic patients compared to 14.4%, 8.4% and 5.7% of non-diabetic patients, respectively), as well as higher long-term mortality [172]. In a 10-year follow-up study of diabetic patients, the risk of mortality was numerically higher with PCI compared with CABG at 5 years [19.6% vs. 13.3%, hazard ratio (HR): 1.53, 95% CI: 0.96, 2.43, *p*  =  0.075], with the opposite seen between 5 and 10 years (PCI vs. CABG: 20.8% vs. 24.4%, HR: 0.82, 95% CI: 0.52, 1.27, *p*  =  0.366) [173]. These findings added to the uncertainty regarding the performance of PCI in patients with diabetes.

Small-vessel disease also remains a major limitation, with multiple studies showing substantially higher restenosis rates in vessels <2.8 mm. In classic angiographic series, restenosis rate was 38.6% in vessels <2.8 mm vs. 20.4% in vessels >3.2 mm [174], and later analyses on DES continued to show greater late lumen loss and higher rates in small compared with large vessels [175]. A similar challenge also exists in bifurcations. A bifurcation lesion is a coronary artery narrowing occurring adjacent to, and/or involving, the origin of a significant side branch. Even with contemporary DES, bifurcation PCI shows worse outcomes than non-bifurcation PCI, with target lesion failure rates typically 10–12% at 1 year, compared with approximately 5–7% for simpler lesions [176]. Chronic total occlusions, characterized by long lesion length, heavy calcification, and tortuosity, pose difficulties in the choice of intervention technologies. According to a randomized trial, the composite rate of cardiac death, MI or target lesion failure alters from 12% for intravascular ultrasound-guided intervention compared to 27% for angiography-guided [177]. Finally, patients at high bleeding risk represent an unresolved dilemma. Trials such as LEADERS FREE and MASTER-DAPT have shown that a 1-month DAPT regimen with modern DES can reduce bleeding. However, patients at high bleeding risk continue to face substantially elevated risks. Their likelihood of both bleeding and ischemic complications remains nearly twofold higher compared with patients without high bleeding risk [178,179]. Collectively, these data demonstrate that even with state-of-the-art DES, complex anatomic and clinical subsets continue to experience unsatisfactory outcomes, enhancing the need for technological innovation in PCI.

As stent technology continues to evolve, regulatory, manufacturing, and real-world adoption challenges are also escalating. First of all, as combination products (metal scaffold, polymer coating, drug), coated stents are subject to complex regulatory oversight. For example, the FDA requires extensive non-clinical testing of mechanical integrity, polymer stability, drug-elution kinetics, and chronic biocompatibility before market approval [180], which could take years. Secondly, manufacturing consistency is difficult: ensuring uniform polymer coating, reproducible drug loading, and durability under real-world deployment (balloon expansion, vessel curvature, cyclic stress) is technically demanding, and small deviations across batches may compromise safety or efficacy [181]. Additionally, post-market adoption in clinical practice varies between hospitals, areas and countries. In the U.S., although DES is reported to be used in >75% of PCI patients overall [182], only 49% of PCI cases in Arkansas used a DES, with rates dropping down to 35% for rural hospitals [183]. In middle and low-income countries, economic constraints and lack of reimbursement remain major barriers to DES use [184].

## 7. Limitations

This narrative review is subject to several limitations. Because it does not follow a systematic search process, selection bias is possible, and some relevant studies may have been missed. Standards used to classify stent generations in this study are not universally acknowledged, which may affect cross-study comparisons. Evidence for newer technologies, such as biodegradable alloys or smart coatings, is largely based on preclinical or data, limiting certainty regarding long-term clinical outcomes. Regional variations in stent adoption and resource availability may also restrict generalizability. Finally, rapid innovation in stent technologies means that emerging data may refine or alter some conclusions after publication.

## 8. Conclusions

Coronary stent development has transformed PCI by reducing acute complications, thrombosis, and restenosis. These advances have led to significantly improved clinical outcomes and represent a major achievement in engineering and drug delivery. Nonetheless, important challenges remain. Late safety issues with first-generation DES, modest gains of newer DESs in complex patients, and barriers related to regulation, manufacturing, cost, and global access continue to limit progress. Future progress will require stents that support faster and more complete vascular healing, perform reliably in complex anatomies, and balance long-term durability with biocompatibility. Equally important are robust long-term clinical evaluations and strategies that broaden access across diverse healthcare settings. These efforts will guide the development of safer, more effective, and more equitable coronary interventions.

## Figures and Tables

**Table 1:** Complications, overall incidence rate, and outcomes after SS-based BMS implantation.

Complication	Incidence Rate	Outcomes	Reference
Stent Thrombosis	12–20% (no DAPT) <1–4% (with DAPT)	High morbidity and mortality; often leads to MI or sudden cardiac death.	[33,39,40,42,43,44]
In-Stent Restenosis	17–41% (SS-based BMS) 2.5–5% (New generation DES)	Typically presents as recurrent angina; may require repeat revascularization procedures.	[36,37,38]
Bleeding	2–3%	Increased risk of hemorrhagic events, including gastrointestinal bleeding and anemia.	[45,46]
Stent Infection	<0.1%	High mortality; often requires urgent intervention but is frequently fatal.	[47,48,49]
Coronary Artery Dissection	1–2%	Can lead to acute vessel closure; severe cases may require emergency CABG.	[50]
Late Stent Malapposition	4–5%	Potential cause of late restenosis or thrombosis; may necessitate further intervention.	[51,52]

**Table 2:** Examples of first- to fourth-generation DES and their main parameters. SS: stainless steel; CoCr: cobalt-chromium; PtCr: platinum-chromium; PLLA: poly(l-lactide); PEVA: poly(ethylene-co-vinyl acetate); PBMA: poly(n-butyl methacrylate); SIBS: styrene-isobutylene-styrene; PLGA: poly(lactic-co-glycolic acid); PDLLA: poly(d,l-lactic acid). Late lumen loss is a key indicator of stent performance, measuring the degree of vessel narrowing after stent implantation, and is normally assessed at 6 to 9 months.

Generation	Name	Manufacturer	Material	Strut Thickness (µm)	Polymer Type	Polymer Thickness (µm)	Drug	Drug Release Kinetics	Late Lumen Loss (mm)
1st	Cypher	Cordis	SS	140	Durable (PEVA/PBMA)	12.6	Sirolimus	80% in 30 days	0.17–0.24 [84]
	Taxus	Boston Scientific	SS	132	Durable (SIBS)	16	Paclitaxel	<10% in 30 days	0.23–0.39 [94]
2nd	Xience V	Abbott (Chicago, IL, USA)	CoCr	81	Durable (Fluoropolymer)	7.6	Everolimus	Full release in 90 days	0.12–0.17 [95]
	Resolute	Medtronic (Galway, Ireland)	CoCr	91	Durable/ Biodegradable (BioLinx)	4.1	Zotarolimus	Gradual release in 6 months	Mean 0.12 [96]
3rd	Synergy	Boston Scientific	PtCr	74	Biodegradable (PLGA)	4	Everolimus	Full release in 90 days	Mean 0.10 [97]
	Orsiro	Biotronik (Berlin, Germany)	CoCr	60	Biodegradable (BIOlute)	7.5	Sirolimus	50% in 30 days	Median 0.06 [96]
4th	Absorb	Abbott	PLLA	130–150	Fully bioresorbable (PDLLA)	/	Everolimus	80% in 30 days	Mean 0.37 [98]

## Data Availability

All data are publicly available on the MEDLINE/Pubmed platforms.

## Abbreviations

ACC/AHA	American college of cardiology/American heart association
ACS	acute coronary syndromes
BMS	bare-metal stent
BVS	bioresorbable vascular scaffold
CAD	coronary artery disease
CABG	coronary artery bypass grafting
CI	confidence interval
CoCr	cobalt-chromium
DAPT	dual antiplatelet therapy
DBCO	dibenzocyclooctyne
DES	drug-eluting stent
EMA	European medicines agency
ESC	European society of cardiology
FDA	U.S. food and drug administration
HR	hazard ratio
ISR	in-stent restenosis
LST	late stent thrombosis
MI	myocardial infarction
NIR	near-infrared
NO	nitric oxide
PBMA	poly(n-butyl methacrylate)
PCI	percutaneous coronary intervention
PDA	polydopamine
PDLLA	poly(d,l-lactic acid)
PEVA	poly(ethylene-co-vinyl acetate)
PLGA	poly(lactic-co-glycolic acid)
PLLA	poly(l-lactide)
PtCr	platinum-chromium
ROS	reactive oxygen species
RR	risk ratio
SIBS	styrene-isobutylene-styrene
SMC	smooth muscle cell
SS	stainless steel
ST	stent thrombosis
